# Copper hydroxychloride is more efficacious than copper sulfate in improving broiler chicken's growth performance, both at nutritional and growth-promoting levels

**DOI:** 10.1016/j.psj.2020.09.053

**Published:** 2020-10-09

**Authors:** H.T.T. Nguyen, N. Morgan, J.R. Roberts, R.A. Swick, M. Toghyani

**Affiliations:** ∗ Department of Animal Science, School of Environmental and Rural Science, University of New England, Armidale, NSW 2351, Australia; † Faculty of Science, School of Life and Environmental Sciences, The University of Sydney, Sydney NSW 2006, Australia

**Keywords:** broiler, copper hydroxychloride, performance, tibia, tissue mineral

## Abstract

This study was designed to compare the effects of nutritional and growth-promoting levels of copper hydroxychloride (**CH**) with copper sulfate (**CuSO_4_**) on growth, carcass characteristics, tibia traits and mineral concentration in broilers fed a conventional wheat-soybean meal–based diet. Day-old Ross 308 male chicks (*n* = 864) were randomly assigned into 8 dietary treatments with 6 replicates of 18 chicks per treatment. The dietary treatments included a basal diet containing no supplemental copper (**Cu**) serving as the negative control (**NC**); basal diet supplemented with 15 or 200 mg/kg Cu as CuSO_4_; basal diet supplemented with either 15, 50, 100, 150, or 200 mg/kg Cu from CH. Diets were fed over the starter (day 1–14) and grower (day 14–35) phases. Birds in the NC group gained the same body weight and had similar feed conversion ratio (**FCR**) to birds receiving 15 mg/kg Cu as CuSO_4_, but birds receiving 15 mg/kg Cu as CH had a lower FCR than the NC birds (day 0–35; *P* < 0.05). Birds fed 200 mg/kg Cu as CH gained more weight (77 g/bird) and had a lower FCR (3.2 point) compared with those fed 200 mg/kg Cu as CuSO_4_ (*P* < 0.01). Based on broken-line regression models, the optimum inclusion level of Cu as CH in the diet for optimal body weight gain and FCR were estimated to be 109.5 and 72.3 mg/kg, respectively (*P* < 0.001). Carcass characteristics were not affected by dietary Cu sources or levels (*P* > 0.05). The highest and lowest tibia ash content were observed in birds fed diet with 150 mg/kg Cu as CH and 200 mg/kg Cu as CuSO_4_, respectively (*P* < 0.05). Supplementation with 200 mg/kg Cu as CH resulted in higher duodenal mucosa Cu content compared with the diet containing 200 mg/kg Cu as CuSO_4_ (*P* < 0.001). In conclusion, supplementation of Cu from CH was more efficacious than CuSO_4_ in promoting growth performance, both at nutritional and pharmacological levels.

## Introduction

Antibiotics have long been used at subtherapeutic levels as antibiotic growth promoters (**AGP**) in poultry production. However, the risk of bacteria becoming resistant to certain antibiotics has led the poultry industry to look for alternatives to AGP possessing antimicrobial properties which can maintain intestinal health and improve growth performance (Toghyani et al., 2010). Copper (**Cu**) has been reported to have antimicrobial properties when used at doses higher than the required nutritional levels (Fisher et al., 1973; Pesti and Bakalii, 1996; Wanga et al., 2008), resulting in improved body weight gain (**BGW**) and feed efficiency in broiler chickens.

Copper is an essential trace element for various enzymatic systems involved in oxidative and reductive processes, such as cytochrome oxidase, lysyl oxidase, superoxide dismutase, ceruloplasmin, and metallothionein (Lim and Paik, 2006). The recommended nutritional requirement of Cu in broiler diets is in the range from 8 mg/kg (NRC, 1994) to 15 mg/kg (Aviagen, 2014). Reports on the efficacy of Cu being used at pharmacological levels, as an alternative to AGP in poultry diets, are inconsistent. For example, Nys (2001) suggested that a high concentration of Cu (100–250 mg/kg) in the feed tends to slightly improve growth performance, whereas Banks et al. (2004) and Pang and Applegate (2007) did not observe any significant effects on birds growth performance when Cu was supplemented at levels excess to the requirements (up to 250 mg/kg). Nonetheless, Cu included at levels over 250 mg/kg has been shown to negatively affect productive traits (Pesti and Bakalii, 1996).

The stability and biological availability of supplemental Cu sources are of utmost importance, particularly when it is applied at pharmacological levels. In common industry practice, Cu is added in ionic forms such as Cu sulfate (**CuSO_4_**). Sulfate molecules are highly reactive in the presence of moisture and can easily break down both in the feed and the upper gastrointestinal tract, releasing reactive free Cu ions which can bind other dietary nutrients such as vitamins, fats, and enzymes, consequently hindering the bioavailability of both Cu and such nutrients (Close, 1998).

Copper hydroxychloride (**CH**) is an inorganic source of Cu, which is less soluble in water but is soluble in acidic solutions when compared with Cu sulfate (Cromwell et al., 1998; Pang and Applegate, 2007). Copper hydroxychloride is formed by strong covalent bonds between Cu, the hydroxy groups, and chloride ions. This crystallized molecular structure makes CH less reactive in catalyzing the destruction of certain vitamins and organic compounds when concentrated in base mixes or in supplements and diets (Cromwell et al., 1998). Compared with Cu sulfate, the pro-oxidant activity of CH has been reported to be lower (Miles et al., 1998; Hooge et al., 2000). For example, Luo et al. (2005) indicated that CH is chemically more stable than Cu sulfate and does not promote the oxidation of feed vitamin E.

Trace minerals play a significant role in the growth and development of the skeletal system of farm animals, in particular in fast-growing chickens (Julian, 2005). The assimilability of such elements can affect the growth and strength of bones (Dibner et al., 2007). For example, Cu is a cofactor for the enzyme lysyl oxidase, which participates in the cross-linking of both collagen and elastin proteins and is critical for tissue structural integrity (Rucker et al., 1998). Consequently, copper deficiency results in increased cell permeability and reduced collagen strength (Kwiecień et al., 2014). Thus, the extent of bone mineralization could be considered as a robust indicator of the overall body reserves of trace minerals and the quality of mineral premixes (Crespo et al., 2000). The main objective of the present study was to compare the efficacy of different levels of CH with CuSO_4_ on performance, tibia-breaking strength, and mineralization of broiler chickens fed a wheat-soybean meal diet. Bird growth responses to Cu added as CH were also used to determine the optimum level of CH in the diet for maximum BGW and feed efficiency.

## Materials and methods

All the experimental procedures used in this study were reviewed and approved by the University of New England Animal Ethics Committee.

### Birds and Housing

A total of 864 male day-old Ross 308 chicks were transported from Aviagen, Goulburn, NSW, Australia, to the Centre for Animal Research and Teaching at the University of New England. On arrival, chicks were weighed and randomly assigned to 48 floor pens. Each pen measured 1.2 m × 0.75 m and was equipped with a tube feeder and 2 cup drinkers with fresh hardwood shavings as bedding material. Room temperature was maintained at 34°C during the first 3 d, then gradually reduced to 23°C at the end of week 3, and kept constant thereafter. The lighting program and ventilation followed the recommendations of the Ross 308 breed management manual (Aviagen, 2014). Birds had *ad libitum* access to water and feed throughout the entire study.

### Experimental Treatments and Design

The birds were randomly assigned into 8 dietary treatments, each replicated 6 times, with 18 chicks per replicate. The dietary treatments met the nutrient specifications of the strain, as recommended by the Ross 308 guidelines (Aviagen, 2014 - Table 1). The 8 dietary treatments comprised a negative control diet (the basal diet—NC) without any supplemental Cu, a basal diet supplemented with either 15 (as a nutritional dose) or 200 mg/kg (as a growth-promoting dose) Cu as CuSO_4_, and 5 diets supplemented with 15 (as a nutritional dose), 50, 100, 150, or 200 mg/kg (as growth-promoting doses) Cu as CH (Selko IntelliBond Cu, Trouw Nutrition, the Netherlands). The broiler chicks received the wheat-soybean meal–based experimental diets in 2 phases from day 1 to 14 (starter) and day 14 to 35 (grower). All pens were checked for mortality twice daily and feed intake (**FI**) was corrected for mortality for each period.

**Table 1:** Diet composition and nutritive value of the experimental diets (as-fed basis).

Ingredients, %	Starter (day 1–14)	Grower (day 14–35)
Wheat	53.79	55.77
Soybean meal	27.71	19.85
Canola meal	9.74	12.80
Rice bran	4.73	6.29
Canola oil	1.56	3.07
Limestone	1.19	1.18
Dicalcium phosphate^1^	0.28	0.05
Salt	0.17	0.12
Na bicarb	0.10	0.13
Mineral premix^2^	0.10	0.10
Vitamin premix^3^	0.09	0.09
Choline Cl 60%	0.05	0.05
L-lysine HCl 78.4	0.23	0.24
D,L-methionine	0.20	0.17
L-threonine	0.04	0.05
Xylanase	0.02	0.02
Phytase	0.01	0.01
Total	100.0	100.0
Calculated nutrients		
ME, kcal/kg	3,000	3,150
Crude protein %	23.86	21.62
Crude fat %	4.62	6.64
Crude fiber %	3.62	3.85
d Arg %	1.34	1.15
d Lys %	1.24	1.10
d Met %	0.53	0.48
d M + C %	0.90	0.83
d Trp %	0.28	0.24
d Ile %	0.89	0.78
d Thr %	0.79	0.72
d Val %	0.98	0.88
Calcium %	0.85	0.80
Available phosphorus %	0.43	0.40

1 Dicalcium phosphate contained: phosphorus, 18%; calcium, 21%.

2 The Cu-free trace mineral concentrate supplied per kilogram of diet: Zn (sulfate), 60 mg; Fe (sulfate), 40 mg; I (iodide), 1.5 mg; Se (selenate), 0.3 mg; Mn (sulfate), 80 mg; millrun-based carrier, 128 mg; mineral oil, 100 mg.

3 Vitamin concentrate supplied per kilogram of diet: retinol, 12,000 IU; cholecalciferol, 5,000 IU; tocopheryl acetate, 75 mg, menadione, 3 mg; thiamine, 3 mg; riboflavin, 8 mg; niacin, 55 mg; pantothenate, 13 mg; pyridoxine, 5 mg; folate, 2 mg; cyanocobalamin, 16 μg; biotin, 200 μg; cereal-based carrier, 149 mg; mineral oil, 2.5 mg.

### Data Collection

Performance parameters including FI, BGW, feed conversion ratio (**FCR**), and livability rate were measured for the starter (1–14 d), grower (14–35 d), and the entire experimental period (1–35 d).

On day 14, 3 birds in each pen, representing pens mean body weight, were selected and stunned, then euthanized by decapitation to collect blood in vacutainer tubes (BD, Wokingham, Berkshire, UK). Blood samples were centrifuged at 3,000 × *g* for 10 min at 4°C, following 30 min clotting at room temperature to separate the serum, then pooled into individual Eppendorf tubes and subsequently frozen at −20°C, before analysis for Cu concentration. The right tibia was collected, and all soft tissue and cartilage removed; then the tibia bone was stored in a refrigerator before the measurement of tibia bone traits. A section of the duodenum was obtained and washed with distilled water, then opened longitudinally. The duodenal mucosa was scraped gently using a microscope slide, and this mucosa was pooled within replicate groups into plastic containers. Duodenal mucosa samples were stored at −20°C for mineral concentration analysis.

**Figure 1:**
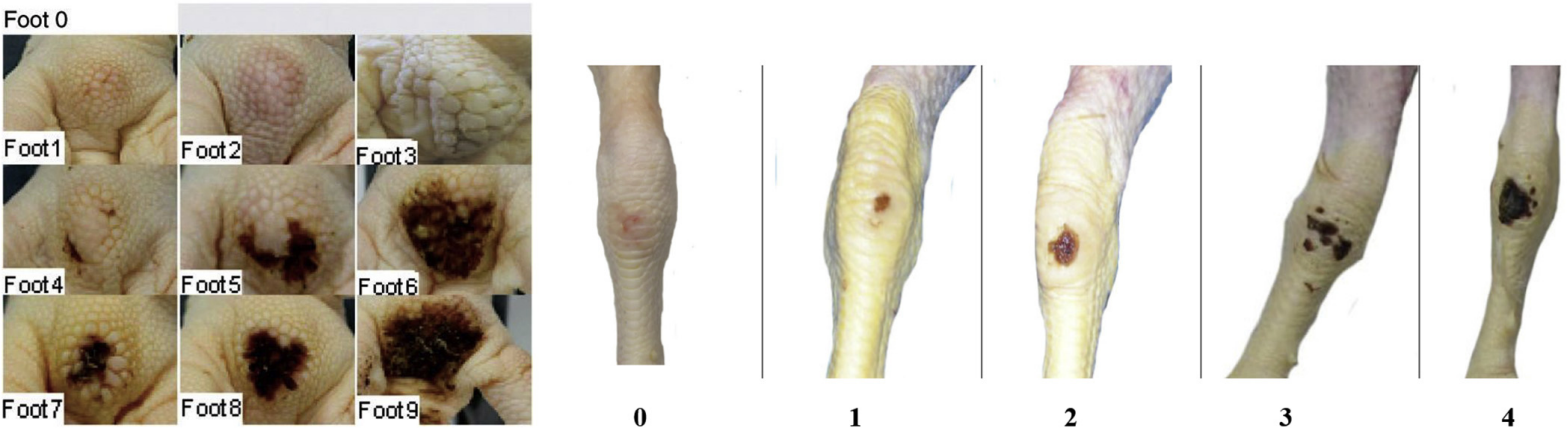
Scoring system for footpad lesions (left) and hock lesions (right) based on visual observations.

On day 35, 3 randomly selected birds were slaughtered to collect the right tibia and to measure carcass traits. The tibiae were collected after the procedure applied on day 14, and all tibiae were used to measure breaking strength and to determine ash content and mineral concentration. Carcass parts including the breast meat, thigh, fat pad, liver, gizzard, pancreas, bursa, and spleen were removed and weighed, and weights were expressed as g/100 g live body weight. In addition, the same birds were used to evaluate footpad dermatitis and hock burns (lesions) on both feet. Any signs of footpad dermatitis were scored from 0 to 9 for footpads (Allain et al., 2009) and 0 to 4 for hock burns (Welfare Quality, 2009), based on visual observation (Figure 1).

### Chemical Analysis and Tibia-Breaking Strength Test

The mineral content in the diets and premixes, tibia, and duodenal mucosa was determined using an inductively coupled plasma–optical emission spectrometer (**ICP-OES**) (Agilent, Mulgrave, Victoria, Australia).

Triplicate representative composite samples from each diet and mineral premix (as-fed basis) were collected and ground into fine particles (0.5 mm) for analysis of Cu concentration in duplicate. Then 0.5 g of each diet sample was weighed into white Teflon tubes (Milestone, Sorisole, Bergamo, Italy) and then digested in 1 mL distilled water and 4 mL concentrated HCl (70%) in an Ultrawave Microwave Digestion system (Milestone, Sorisole, Bergamo, Italy). The digested samples were diluted with distilled water to a volume of 25 mL in a 30 mL volumetric flask for analysis of mineral concentration by ICP-OES instrument.

The tibiae were subjected to breaking strength using an Instron instrument (LX 300 Instron, Norwood, MA) set up with a 300 kN load cell and 3 point fixture bed, at a test speed of 10 points of data per second and run using Blue Hill 3 software. The tibia bones were then dried at 105°C for 24 h in a drying oven (Qualtex Universal Series 2000, Watson Victor Ltd., Perth, Australia) to determine dry mass, and ashed in a chamber furnace (Carbolite CWF 1200, Sheffield, UK) at 600°C for 6 h. Then 0.1 g of each ash sample was weighed for analysis of mineral concentration by ICP-OES technique (as described above). The tibia mineral content was calculated as a proportion of the crude ash.

Duodenal mucosal samples were freeze-dried at −50°C for 1 wk in a freeze-drying system (Alpha 1-4 LDplus, Christ, Osterode am Harz, Germany) and passed through a 0.5 mm screen. Then 0.5 g of dried mucosa sample was weighed for analysis of Cu concentration by the ICP-OES technique.

Serum Cu concentration was determined colorimetrically using a commercial kit (Colorimetric Method Manual RX Monza, Randox Laboratories, Crumlin, County Antrim, UK), in accordance with the manufacturer's protocol, followed by running the plate reader in a spectrophotometer (SpectraMax M2e, Molecular Devices, San Jose, CA).

### Statistical Analysis

All data were checked for normal distribution before conducting statistical analysis using the UNIVARIATE procedure of SAS 9.3 (SAS Institute Inc., 2010). Data were then subjected to one-way ANOVA analysis in a completely randomized design using the General Linear Model procedure of SAS 9.3. Nonparametric analysis (PROC NPAR1WAY WILCOXON) was performed on livability, hock, and footpad lesions data. Each single pen was considered as an experimental unit, and the values presented in the tables are means with pooled SEM (*n* = 48). Tukey's HSD test was used to make pairwise comparisons between means, when a significant effect of treatment was detected. Significant values are based on *P* ≤ 0.05, with a trend suggested at *P* > 0.05 but <0.10. Orthogonal contrasts were made to detect statistical significances between treatment means of the 200 mg/kg CuSO_4_–supplemented group vs. CH–supplemented groups, and the NC group vs. 15 mg/kg CuSO_4_– and 15 mg/kg CH–supplemented groups. The optimum Cu levels from CH for the optimal weight gain and feed efficiency was determined using linear broken-lines regression analysis following the method of Pesti et al. (2009).

## Results

The analyzed Cu concentration in the basal diet (**NC**) was 9.1 mg/kg for the starter phase and 7.6 mg/kg for the grower phase (Table 2). The analyzed Cu values in other diets were consistently higher than the calculated values by an average of 10 to 15 mg/kg.

**Table 2:** The analyzed Cu concentration in the mineral premixes and dietary treatments.^1^

Treatments^2^	Cu source^2^	Cu added mg/kg	Premix g/kg	Calculated Cu mg/kg		Analyzed Cu mg/kg	
				Starter	Grower	Stater	Grower
1—NC	None	0	0	8	8	9.1	7.6
2	CuSO_4_	15	15.1	23	23	27.3	24.8
3	CuSO_4_	200	209.1	208	208	223.2	222.4
4	CH	15	16.4	23	23	26.1	25.6
5	CH	50	57.5	58	58	68.7	59.7
6	CH	100	116.9	108	108	116.5	110.3
7	CH	150	158.1	158	158	164.0	165.2
8	CH	200	203.7	208	208	215.3	230.3

1 Values based on chemical analysis of duplicate samples of diets and reported on an as-fed basis.

2 NC, negative control, no added Cu; CH, copper hydroxychloride (IntelliBond Cu).

Table 3 summarizes the performance parameters of broilers in response to the dietary treatments. The birds fed the NC and 15 mg/kg CuSO_4_ diets gained the least weight and had the highest FCR among all the treatments (*P* < 0.01; 1–35 d). The greatest BWG and lowest FCR were observed in birds fed the 200 mg/kg CH treatment, followed by the 100 mg/kg CH group (*P* < 0.01). Birds receiving 200 mg/kg CH had statistically higher weight gain (77 *g*) and lower FCR (3.2 points) compared with the birds receiving CuSO_4_ at 200 mg/kg (*P* < 0.01), as revealed by orthogonal contrast comparisons. No significant differences in FI were detected in either the starter or grower periods (*P* > 0.05). Orthogonal contrast analysis did not reveal any statistical differences between birds receiving NC with 15 mg/kg Cu as CuSO_4_ or 15 mg/kg as CH for any parameters measured during the starter or grower periods (*P* > 0.05). However, over the entire production period, birds in the 15 mg/kg CH group had lower FCR than the NC group (*P* = 0.013). Dietary inclusion of 15 mg/kg CH vs. 15 mg/kg CuSO_4_ resulted in higher final BWG and lower FCR (*P* < 0.05). Supplementation of 100 or 150 mg/kg CH compared with 200 mg/kg CuSO_4_ did not affect BWG but significantly improved FCR during the grower and entire production periods (*P* < 0.001).

**Table 3:** Growth performance parameters of broilers in response to different dietary treatments.

Treatments^1^	Starter (day 1–14)				Grower (day 14–35)			Overall (day 1–35)			
	IBW, g	BWG, g	FI, g	FCR^2^	BWG, g	FI, g	FCR^2^	BWG, g	FI, g	FCR^2^	Livability, %
(1) NC	42.0	456	554	1.213^a^	2,002^b,c^	3,004	1.501^a^	2,458^c^	3,558	1.447^a^	100.0
(2) CuSO_4_ 15 mg/kg	41.9	464	556	1.197^a,b^	1,985^c^	2,943	1.482^a^	2,450^c^	3,500	1.428^a^	96.7
(3) CuSO_4_ 200 mg/kg	41.8	470	548	1.167^b,c^	2,036^a,b,c^	2,951	1.451^b^	2,504^b,c^	3,501	1.397^b^	98.9
(4) CH 15 mg/kg	42.4	472	547	1.159^c^	2,046^a,b^	2,955	1.444^b,c^	2,518^a,b,c^	3,502	1.391^b,c^	100.0
(5) CH 50 mg/kg	41.6	475	546	1.150^c^	2,040^a,b^	2,939	1.441^b,c^	2,514^a,b,c^	3,486	1.386^b,c^	97.8
(6) CH 100 mg/kg	42.2	485	556	1.146^c^	2,073^a,b^	2,962	1.429^b,c^	2,558^a,b^	3,519	1.375^b,c^	100.0
(7) CH 150 mg/kg	41.7	480	553	1.152^c^	2,074^a,b^	2,970	1.432^b,c^	2,554^a,b^	3,523	1.379^b,c^	97.8
(8) CH 200 mg/kg	41.9	492	562	1.142^c^	2,088^a^	2,963	1.419^c^	2,580^a^	3,525	1.365^c^	97.8
SEM^3^	0.19	8.1	10.2	0.007	16.9	30.3	0.006	16.6	29.5	0.005	1.08
*P*-value	0.122	0.072	0.956	0.001	0.009	0.869	0.001	0.001	0.769	0.001	0.221

Orthogonal contrasts	Probabilities										
(1) vs. (2)	NS	NS	NS	NS	NS	NS	NS	NS	NS	NS	NS
(1) vs. (4)	NS	NS	NS	NS	NS	NS	NS	NS	NS	0.013	NS
(2) vs. (4)	NS	NS	NS	NS	NS	NS	NS	0.050	NS	0.041	NS
(3) vs. (5)	NS	NS	NS	NS	NS	NS	NS	NS	NS	NS	NS
(3) vs. (6)	NS	NS	NS	NS	NS	NS	0.001	NS	NS	<0.001	NS
(3) vs. (7)	NS	NS	NS	<0.001	NS	NS	<0.001	NS	NS	<0.001	NS
(3) vs. (8)	NS	NS	NS	0.006	0.048	NS	0.001	0.027	NS	<0.001	NS

^a–d^values in a column with no common superscripts differ significantly (*P* ≤ 0.05). NS, not significant (*P* > 0.05).

Mean values are based on 18 birds per replicate and 6 replicates per treatment.

Abbreviations: BWG, body weight gain; FCR, feed conversion ratio; FI, feed intake; IBW, initial body weight.

1 NC, negative control, no added Cu; CH, copper hydroxychloride (IntelliBond Cu).

2 FCR corrected for mortality.

3 Pooled standard error of means.

Based on fitted broken-line models for performance parameters (Table 4), the predicted optimum levels of supplemental Cu as CH in the diet to achieve optimal BWG and FCR were 109 and 72 mg/kg feed (*P* < 0.001), respectively.

**Table 4:** The optimum levels of dietary supplemental Cu from copper hydroxychloride for broiler chickens as estimated based on fitted broken-line models.^1^

Items	Model parameters^2^			Adj R^2^	CH requirement^3^	Asymmetric SE	Asymmetric 95% Cl^4^
1–14 d							
BWG, g/b/d	L = 34.7	U = −0.0233	R = 77.4	0.22	77.4	0.338	8.2–163.1
P-value	0.0001	0.1582	0.0859				
FCR, g/g	L = 1.143	U = 0.0006	R = 79.3	0.43	79.3	0.018	23.5–135.1
P-value	0.006	0.0001	0.0087				
14–35 d							
BWG, g/b/d	L = 99.0	U = −0.027	R =113.7	0.38	113.7	0.478	50.5–176.9
P-value	0.0001	0.0045	0.0013				
FCR, g/g	L = 1.424	U = 0.0008	R = 72.3	0.59	72.3	0.013	39.2–105.5
P-value	0.0001	0.0001	0.0001				
1–35 d							
BWG, g/b/d	L = 73.3	U = −0.0232	R =109.5	0.53	109.5	0.298	65.3–153.7
P-value	0.0001	0.0029	0.0001				
FCR, g/g	L = 1.371	U = 0.0008	R = 72.3	0.61	72.3	0.005	40.2–104.5
P-value	0.0001	0.0001	0.0001				

Abbreviations: Adj, adjusted R-square; BWG, body weight gain; FCR, feed conversion ratio.

1 Defined as the breaking points of broken-line models of the selected variables as calculated by a nonlinear least squares analysis.

2 In the model, Y (output) = L (Max/Min) + U (Slope ratio) × (Requirement–X); where L is value at the breaking point, U = slope ratio of line at X < R and R = value of X at the breaking point. The estimates of this model are valid if X < R; when X ≥ R, then Y = Max/Min.

3 CH, copper hydroxychloride (IntelliBond Cu).

4 Cl, confidence intervals (lower and upper 95% confidence levels).

In accordance with the results presented in Table 5, the relative weight of the carcass, other internal organs, footpad dermatitis and hock burn scores on day 35 after hatch were not affected by Cu sources or the levels in the diet (*P* > 0.05).

**Table 5:** Carcass traits and foot scores of broilers response in the dietary treatments recorded at day 35.

Treatments^1^	Carcass traits (g/100 g live body weight)								Foot score	
	Breast	Thigh	Fat pad	Liver	Gizzard	Pancreas	Bursa	Spleen	Footpad	Hock burn
(1) NC	22.1	17.5	0.63	2.34	1.21	0.185	0.134	0.066	1.33	1.66
(2) CuSO_4_ 15 mg/kg	21.7	18.0	0.64	2.46	1.22	0.188	0.128	0.072	0.83	2.33
(3) CuSO_4_ 200 mg/kg	22.3	17.9	0.54	2.32	1.16	0.167	0.123	0.073	1.50	1.83
(4) CH 15 mg/kg	22.1	17.8	0.56	2.55	1.31	0.197	0.131	0.072	0.33	2.33
(5) CH 50 mg/kg	21.8	18.0	0.65	2.47	1.21	0.188	0.135	0.072	0.16	1.66
(6) CH 100 mg/kg	21.5	17.7	0.55	2.53	1.19	0.195	0.136	0.072	0.66	1.33
(7) CH 150 mg/kg	21.8	18.0	0.58	2.45	1.22	0.179	0.145	0.082	0.66	1.33
(8) CH 200 mg/kg	22.2	17.8	0.62	2.33	1.16	0.177	0.139	0.075	0.83	1.50
SEM^2^	0.419	0.192	0.046	0.088	0.071	0.009	0.006	0.005	0.751	0.599
*P*-value	0.498	0.682	0.503	0.416	0.873	0.349	0.202	0.766	0.918	0.870

Orthogonal contrasts	Probabilities									
(1) vs. (2)	NS	NS	NS	NS	NS	NS	NS	NS	NS	NS
(1) vs. (4)	NS	NS	NS	NS	NS	NS	NS	NS	NS	NS
(2) vs. (4)	NS	NS	NS	NS	NS	NS	NS	NS	NS	NS
(3) vs. (5)	NS	NS	NS	NS	NS	NS	NS	NS	NS	NS
(3) vs. (6)	NS	NS	NS	NS	NS	NS	NS	NS	NS	NS
(3) vs. (7)	NS	NS	NS	NS	NS	NS	NS	NS	NS	NS
(3) vs. (8)	NS	NS	NS	NS	NS	NS	NS	NS	NS	NS

Mean values are based on 3 birds per replicate and 6 replicates per treatment; NS, not significant (*P* > 0.05).

1 NC, negative control, no added Cu; CH, copper hydroxychloride (IntelliBond Cu).

2 Pooled standard error of means.

Data on tibia-breaking strength and mineral composition determined on day 14 and 35 are presented in Table 6. Dietary treatments tended (*P* = 0.059) to influence tibia-breaking strength on day 35 of age, with birds in NC and 200 mg/kg CH–supplemented diets recording the lowest and the highest breaking strength values, respectively. A significant effect (*P* < 0.05) of the treatments on tibia bone ash was detected only on day 14, where birds in the 150 mg/kg CH group recorded the highest ash percentage (*P* < 0.05). Diet supplementation with CH at 200 mg/kg resulted in a significant increase (*P* < 0.05) in tibia bone Cu content compared with the NC diet on day 14. Birds fed the low Cu diets, including the negative control and diets with CH and CuSO_4_ at 15 mg/kg, deposited less Mn (μg/g) in the tibia on day 35 (*P* < 0.01). The concentration of other minerals in the tibia, including Zn, Ca, and P, were not significantly affected by the dietary treatments (*P* > 0.05).

**Table 6:** Tibia characteristics and mineral concentration of broilers response to the dietary treatments at day 14 and 35.

Treatments^1^	Breaking strength (N/mm^2^)		Ash (%)		Cu (μg/g)		Zn (μg/g)		Ca (%)		Mn (μg/g)		P (%)	
	Day 14	Day 35	Day 14	Day 35	Day 14	Day 35	Day 14	Day 35	Day 14	Day 35	Day 14	Day 35	Day 14	Day 35
(1) NC	124	285	47.2^a,b^	45.0	3.12^b^	1.91	471	378	34.3	34.9	13.3	11.0^b^	18.1	17.9
(2) CuSO_4_ 15 mg/kg	110	300	47.5^a,b^	45.5	3.30^a,b^	1.87	462	381	33.7	35.1	13.2	10.5^b^	17.9	17.7
(3) CuSO_4_ 200 mg/kg	122	323	47.0^b^	45.5	3.55^a,b^	1.92	468	369	34.3	35.2	13.8	11.7^a,b^	18.1	17.8
(4) CH 15 mg/kg	111	294	47.7^a,b^	45.5	3.67^a,b^	2.00	464	391	34.1	35.1	13.7	10.5^b^	18.3	17.8
(5) CH 50 mg/kg	123	314	47.2^a,b^	45.7	3.59^a,b^	1.93	479	379	34.1	35.1	14.1	11.6^a,b^	18.1	17.7
(6) CH 100 mg/kg	122	328	47.7^a,b^	45.6	3.35^a,b^	1.97	475	374	33.9	35.3	13.2	11.7^a,b^	18.1	17.9
(7) CH 150 mg/kg	112	331	48.6^a^	45.6	3.75^a,b^	1.94	464	372	34.7	35.2	14.7	12.5^a^	18.3	17.7
(8) CH 200 mg/kg	122	338	47.3^a,b^	46.0	3.92^a^	2.00	487	380	34.4	35.3	13.5	11.7^a,b^	18.3	17.9
SEM^2^	8.77	12.99	0.329	0.352	0.161	0.117	10.47	10.51	0.403	0.176	0.663	0.307	0.199	0.109
*P*-value	0.839	0.059	0.041	0.632	0.027	0.966	0.699	0.891	0.758	0.613	0.703	0.004	0.774	0.791

Orthogonal contrasts	Probabilities													
(1) vs. (2)	NS	NS	NS	NS	NS	NS	NS	NS	NS	NS	NS	NS	NS	NS
(1) vs. (4)	NS	NS	NS	NS	NS	NS	NS	NS	NS	NS	NS	NS	NS	NS
(2) vs. (4)	NS	NS	NS	NS	NS	NS	NS	NS	NS	NS	NS	NS	NS	NS
(3) vs. (5)	NS	NS	NS	NS	NS	NS	NS	NS	NS	NS	NS	NS	NS	NS
(3) vs. (6)	NS	NS	NS	NS	NS	NS	NS	NS	NS	NS	NS	NS	NS	NS
(3) vs. (7)	NS	NS	NS	NS	NS	NS	NS	NS	NS	NS	NS	NS	NS	NS
(3) vs. (8)	NS	NS	NS	NS	NS	NS	NS	NS	NS	NS	NS	NS	NS	NS

^a, b^Values in a column with no common superscripts differ significantly (*P* ≤ 0.05); NS, not significant (*P* > 0.05).

Mean values are based on 3 birds per replicate and 6 replicates per treatment.

1 NC, negative control, no added Cu; CH, copper hydroxychloride (IntelliBond Cu). Cu, copper; Zn, zinc; Ca, calcium; Mn, manganese; P, phosphorous.

2 Pooled standard error of means.

Corresponding to the dietary Cu levels, duodenal mucosa Cu content increased with increasing supplemental Cu (*P* < 0.01; Table 7). However, contrast analysis revealed higher Cu in the duodenal mucosa of birds offered diets with Cu from CuSO_4_ at a level of 200 mg/kg compared with those with 50 and 100 mg/kg Cu from CH (*P* < 0.001). The duodenal content of Zn, Mn, Fe, Ca, P, Mg, and Na was not different among the treatments (*P* > 0.05). No significant differences were observed in serum Cu concentration (*P* > 0.05).

**Table 7:** Selected mineral concentration of duodenal mucosa and blood serum copper at day 14.

Treatments^1^	Duodenal mucosa^2^								Serum Cu, μmol/L
	Cu, μg/g	Zn, μg/g	Mn, μg/g	Fe, μg/g	Ca, %	P, %	Mg, %	Na, %	
(1) NC	11.1^d^	123	35.2	419	0.11	1.52	0.142	0.57	3.23
(2) CuSO_4_ 15 mg/kg	11.7^d^	127	35.0	342	0.11	1.56	0.145	0.49	2.61
(3) CuSO_4_ 200 mg/kg	25.3^a^	129	30.7	283	0.12	1.53	0.138	0.47	2.89
(4) CH 15 mg/kg	11.8^d^	120	33.2	259	0.10	1.53	0.142	0.50	3.33
(5) CH 50 mg/kg	15.6^c,d^	126	32.1	443	0.16	1.57	0.141	0.51	3.99
(6) CH 100 mg/kg	19.5^b,c^	126	33.8	384	0.13	1.55	0.141	0.51	3.30
(7) CH 150 mg/kg	23.5^a,b^	125	31.6	426	0.10	1.59	0.143	0.50	3.13
(8) CH 200 mg/kg	27.9^a^	128	30.9	374	0.14	1.55	0.136	0.48	3.22
SEM^3^	1.083	3.34	1.691	69.1	0.028	0.032	0.003	0.020	0.456
*P*-value	0.001	0.700	0.401	0.778	0.796	0.834	0.698	0.621	0.623

Orthogonal contrasts	Probabilities								
(1) vs. (2)	NS	NS	NS	NS	NS	NS	NS	NS	NS
(1) vs. (4)	NS	NS	NS	NS	NS	NS	NS	NS	NS
(2) vs. (4)	NS	NS	NS	NS	NS	NS	NS	NS	NS
(3) vs. (5)	<0.001	NS	NS	NS	NS	NS	NS	NS	NS
(3) vs. (6)	0.005	NS	NS	NS	NS	NS	NS	NS	NS
(3) vs. (7)	NS	NS	NS	NS	NS	NS	NS	NS	NS
(3) vs. (8)	0.088	NS	NS	NS	NS	NS	NS	NS	NS

^a–d^Values in a column with no common superscripts differ significantly (*P* ≤ 0.05); NS, not significant (*P* > 0.05).

Mean values are based on 3 birds per replicate and 6 replicates per treatment.

1 NC, negative control, no added copper; CH, copper hydroxychloride (IntelliBond Cu).

2 Cu, copper; Zn, zinc; Mn, manganese; Fe, iron; Ca, calcium; P, phosphorous; Mg, magnesium; Na, sodium.

3 Pooled standard error of means.

## Discussion

The Cu concentration in the basal diets in this study was close to the minimum requirement (8 mg/kg) recommended for broilers by the NRC (1994). In this study, the comparable productive traits of chicks fed the NC diet (nonsupplemental Cu) with those provided the 15 mg/kg Cu as CuSO_4_ indicates the basal diet based on wheat-soybean meal was not severely deficient in Cu. However, the improvement in BWG and FCR observed with the inclusion of 15 mg/kg Cu as CH indicates the Cu requirements should in fact be more than approximately 8 mg/kg, but most likely CuSO_4_ would have not been as bioavailable as CH. According to Kwiecień et al. (2014), Cu has a stimulating effect on weight gain in chickens due to its participation in the process of hemoglobin synthesis. Copper increases pituitary growth hormone expression (LaBella et al., 1973) and promotes a posttranslational modification of regulatory peptides (Eipper and Mains, 1988). In line with the results of the current trial, previous studies (Cromwell et al., 1998; Luo et al., 2005; M'Sadeq et al., 2018) have also indicated that both broilers and pigs are more responsive to dietary Cu supplementation in the form of CH than CuSO_4_ when assessing performance parameters. This response could be due to stronger covalent bonding between Cu and the other molecules in CH, making Cu less reactive with other nutrients. If CH does not dissolve or dissociate in feed and the upper part of the gastrointestinal tract as quickly as CuSO_4_, this could result in better bioavailability for birds compared with CuSO_4_. For example, Cu supplied in the form of CH may better preserve the stability of organic compounds such as phytase in broiler feed compared with CuSO_4_ (Luo et al., 2005). Hooge et al. (2000) reported that vitamin E recovery was greater in diets containing CH as a supplemental Cu source than those with CuSO_4_ at the same Cu levels.

The use of antibiotics as growth promoters has been banned in many countries (Toghyani et al., 2011). Pharmacological levels of Cu (over 50 mg/kg of diet) above the nutritional requirement (8 mg/kg) are included in broiler chicken diets as a growth promoter (Konjufca et al., 1997; Luo et al., 2005; Pang and Applegate, 2007) because, at pharmacological doses, copper acts as an antimicrobial agent (Fisher et al., 1973; Skrivan et al., 2002) and can favorably affect lipid metabolism (Pesti and Bakalii, 1996). In the present study, when included at pharmacological levels, birds fed 200 mg/kg CH gained more weight and had lower FCR than those receiving CuSO_4_ at 200 mg/kg. This result is in agreement with the findings of Lu et al. (2010), who reported that chicks fed 200 mg/kg Cu as CH had higher weight gain than those fed either lower levels (0–150 mg/kg) of CH or the same level (200 mg/kg) as CuSO_4_. Moreover, the growth-stimulation effects of Cu could be attributed to the changes in the gastrointestinal microbiota, thereby reducing the susceptibility of birds to disease, reducing intestinal lymphocyte recruitment and infiltration, and subsequently increasing nutrient absorption (Arias and Koutsos, 2006; Pang et al., 2009). Another possible reason is that CH modal diameter for particle size was almost 7 times smaller than CuSO_4_ (Miles et al., 1998). A smaller particle has a larger overall surface area, and thus greater exposure to enzymes or proteins for binding and transporting (Pang and Applegate, 2007), which may increase Cu absorption in the gastrointestinal tract and its bacterial penetration. In line with the findings of the present study, Jegede et al. (2011) did not report any significant difference in feed consumption when diets were supplemented with Cu sulfate or Cu proteinate at concentrations of 50, 100, or 150 mg/kg of Cu.

The optimum inclusion levels of supplemental Cu as CH in the wheat-soybean meal–based diet for the best BWG and FCR were predicted at 109 mg/kg and 72 mg/kg of feed, respectively. In a previous study, Arias and Koutsos (2006) reported that the optimal level of supplemental Cu as CH in a corn-soybean diet for growth performance in broiler chicks was 188 mg/kg feed. This discrepancy could be due to the nature of the diets fed, as corn and wheat are different in their macro and micro minerals, intrinsic phytase, and vitamin content (Abed et al., 2018). For instance, wheat contains double the concentration of Cu and Zn compared with corn, whereas corn contains about 4 times more Mn than wheat (Godoy et al., 2005), and has higher phytase and acid phosphatase activities than corn (Viveros et al., 2000). Higher phytase activity in wheat can increase Cu absorption; thus it may partly explain the result of lower estimates of Cu from CH for broilers to optimize growth rate in wheat-based diets compared with corn-based diets.

In the present study, no effect of dietary Cu supplementation was observed on the relative weights of the carcass components, which is similar to the results reported by Hashish et al. (2010). Conversely, Arias and Koutsos (2006) found that broilers offered diets supplemented with 188 mg/kg Cu as CuSO_4_ or CH had higher carcass weights, compared with those fed the negative control diet (8 mg/kg Cu). Olukosi et al. (2018) found that broilers fed CH (15 mg/kg) and Zn hydroxychloride (20 or 80 mg/kg) had greater breast meat yield than those fed CuSO_4_ (15 mg/kg) and Zn sulfate (20 or 80 mg/kg). This may be related to the antimicrobial effects of Cu against pathogenic bacteria, which leads to an increase in nutrient absorption and thus higher protein accretion (Pang and Applegate, 2007).

Copper is a required cofactor for the enzyme lysyl oxidase that is involved in cross-linking of both collagen and elastin proteins (Banks et al., 2004), which in turn increases bone strength. The activity of this enzyme in bone is greatly reduced during Cu deficiency (Siegel et al., 1970). Copper is also a cofactor of antioxidant enzymes, so it could remove bone-free radicals that activate osteoclasts (Kubiak et al., 2010). Copper ions can inhibit osteoclastic resorption but cause lower activation of osteoblasts (Li and Yu, 2007). Therefore, inadequate intake of Cu can result in low bone strength. In this study, the tibia-breaking strength tended to improve with increasing supplemental Cu levels in the diets.

The crude ash content of bones can be a good indicator of the degree of bone mineralization, depending on the availability of Ca, P, and other minerals (Kwiecień et al., 2014). Mikulski et al. (2009) found that tibia ash content and the concentrations of major mineral elements (Ca and P) were unaffected by dietary Cu treatments (20 mg/kg Cu from Cu sulfate or Cu amino acid chelate) in turkeys on day 56. In this study, no effect of dietary Cu on tibia ash content was observed at day 35. It has been reported that pharmacological levels of Cu may compromise dietary phosphorus bioavailability for broilers by forming an insoluble complex of phytate-Cu (Banks et al., 2004). However, the tibia Ca and P content measured in this study indicates that the basal diet may contain enough nonphytate phosphorus to sustain normal bone development and to neutralize the antagonistic effects of the high level of Cu on dietary phosphorus absorption (Hamdi et al., 2018). In addition, the tibia from birds fed the diet containing CH had higher Cu content in comparison with that from birds fed the NC diet but did not differ from those fed CuSO_4_ on day 14, suggesting a similar assimilability of Cu into the bones from these 2 sources when fed at a recommended nutrition dosage for broilers (15 mg/kg; Aviagen, 2014). Copper plays an essential role in the digestion of dietary Ca in broilers. In addition, Pang and Applegate (2007) reported that high dietary Cu inhibits Zn from being digested and absorbed. However, the pharmacological dosages of dietary Cu used in this study did not alter the deposition of Zn, Ca, and P, and did not inhibit deposition of Mn in tibiae, suggesting that a high level of supplemental Cu (up to 200 mg/kg) did not interfere with the digestion, absorption, and incorporation of these minerals into the tibia.

The duodenum is the major site of absorption of Cu in chickens (Hurwitz and Bar, 1965). In the present study, birds fed the NC or diets with 15 mg/kg as either CH or CuSO_4_ had the same duodenal mucosal Cu contents. Fischer et al. (1983) reported that high Zn intake induces a high level of metallothionein in the intestinal mucosa, which has a high binding affinity for Cu. However, there was no change of Zn concentration in the duodenal mucosa in the present study; this may indicate that no interaction between Cu from CH and Zn from Zn sulfate (60 mg/kg supplementation) occurred due to less competition for mineral-binding ligand and mineral uptake sites in the mucosa of the gut. In the study of Huang et al. (2015), weaning pigs fed pharmacological concentrations of Cu (225 mg/kg diet) from CuSO_4_ had higher mucosal Cu concentration in the duodenum compared with those fed CH at the same dose, suggesting that more Cu from CuSO_4_ was taken up by the upper small intestine. Conversely, orthogonal contrasts revealed that mucosa Cu content in birds fed 200 mg/kg Cu as CH tended to be higher than that observed in birds fed 200 mg/kg Cu as CuSO_4_. This may indicate that more Cu from CH was taken up, but not necessarily absorbed into the blood, because intracellular Cu can bind to metallothionein, preventing its absorption until the enterocyte is sloughed off and excreted by the intestine (Bauerly et al., 2005). The unchanged serum Cu levels also further confirm this hypothesis.

## Conclusion

The results obtained in this study indicate that supplementation of copper in the form of CH was more efficacious than copper sulfate in promoting growth performance at both nutritional (15 mg/kg) and pharmacological levels (up to 200 mg/kg), through enhancing growth rate and feed efficiency. The optimum Cu inclusion from CH in the diet for optimal BGW and FCR was estimated to be 109.5 and 72.3 mg/kg feed, respectively. The mineral profile of the tibia and duodenal mucosa suggest that the inclusion of high dietary copper either from CH or copper sulfate up to 200 mg/kg does not compromise the bioavailability and absorption of other minerals. A diet with no supplemental copper may not adversely affect footpad lesions and carcass yield; however, it may compromise tibia-breaking strength particularly in older birds.
